# Substance Use Among Persons with Syphilis During Pregnancy — Arizona and Georgia, 2018–2021

**DOI:** 10.15585/mmwr.mm7203a3

**Published:** 2023-01-20

**Authors:** Jeffrey M. Carlson, Ayzsa Tannis, Kate R. Woodworth, Megan R. Reynolds, Neha Shinde, Breanne Anderson, Keivon Hobeheidar, Aisha Praag, Kristen Campbell, Cynthia Carpentieri, Teri Willabus, Elizabeth Burkhardt, Elizabeth Torrone, Kevin P. O’Callaghan, Kathryn Miele, Dana Meaney-Delman, Suzanne M. Gilboa, Emily O’Malley Olsen, Van T. Tong

**Affiliations:** ^1^Eagle Global Scientific, LLC, Atlanta, Georgia; ^2^Division of Birth Defects and Infant Disorders, National Center on Birth Defects and Developmental Disabilities, CDC; ^3^Arizona Department of Health Services; ^4^Division of STD Prevention, National Center for HIV, Viral Hepatitis, STD, and TB Prevention, CDC; ^5^Chickasaw Nation Industries, Inc., Norman, Oklahoma; ^6^Maricopa County Public Health, Phoenix, Arizona; ^7^Georgia Department of Public Health.

Despite universal prenatal syphilis screening recommendations and availability of effective antibiotic treatment, syphilis prevalence during pregnancy and the incidence of congenital syphilis have continued to increase in the United States (*1*,*2*). Concurrent increases in methamphetamine, injection drug, and heroin use have been described in women with syphilis (*3*). CDC used data on births that occurred during January 1, 2018–December 31, 2021, from two states (Arizona and Georgia) that participate in the Surveillance for Emerging Threats to Pregnant People and Infants Network (SET-NET) to describe the prevalence of substance use among pregnant persons with syphilis by congenital syphilis pregnancy outcome (defined as delivery of a stillborn or live-born infant meeting the surveillance case definition for probable or confirmed congenital syphilis). The prevalence of substance use (e.g., tobacco, alcohol, cannabis, illicit use of opioids, and other illicit, nonprescription substances) in persons with a congenital syphilis pregnancy outcome (48.1%) was nearly double that among those with a noncongenital syphilis pregnancy outcome (24.6%). Persons with a congenital syphilis pregnancy outcome were six times as likely to report illicit use of opioids and four times as likely to report using other illicit, nonprescription substances during pregnancy than were persons with a noncongenital syphilis pregnancy outcome. Approximately one half of persons who used substances during pregnancy and had a congenital syphilis pregnancy outcome had late or no prenatal care. Tailored interventions should address barriers and facilitators to accessing screening and treatment for syphilis among persons who use substances. The need for syphilis screening and treatment should be addressed at any health care encounter during pregnancy, especially among persons who use substances.

SET-NET is a longitudinal surveillance approach established to identify infectious exposures, including syphilis, during pregnancy and monitor health outcomes in pregnant persons and their infants (*4*). In collaboration with CDC, Arizona and Georgia conducted enhanced surveillance for both syphilis in pregnancy and congenital syphilis based on case investigations, medical records, and linkage of laboratory results with vital records. Arizona focused surveillance efforts on Maricopa, Pima, and Yuma counties (approximately 80% of the state’s births); Georgia’s surveillance was statewide. Pregnancies were included if 1) the person met the Council of State and Territorial Epidemiologists (CSTE) case definition* for syphilis (all stages) at any point during pregnancy or 2) the person had a syphilitic stillborn or live-born infant or child who met the CSTE case definition for probable or confirmed congenital syphilis. Substance use during pregnancy, obtained from case investigation interviews or from medical records, included use of tobacco (e.g., cigars, cigarettes, smokeless tobacco, or e-cigarettes), alcohol, cannabis, illicit use of opioids (e.g., prescription opioids not taken as prescribed, fentanyl, or heroin), and other illicit, nonprescription substances (e.g., cocaine, methamphetamines, inhalants, or hallucinogens such as LSD or PCP).

Births that occurred during January 1, 2018–December 31, 2021,^†^ and were reported to CDC as of September 9, 2022, were analyzed to compare the prevalence of any substance use among pregnant persons with syphilis by whether their pregnancy outcome met the surveillance case definition for probable or confirmed congenital syphilis^§^ (congenital syphilis pregnancy outcome) or did not (noncongenital syphilis pregnancy outcome) and to describe selected demographic, prenatal care, clinical and treatment information, and history of incarceration and homelessness in the 12 months preceding case report or positive test results or during pregnancy. All analyses were performed using R statistical software (version 4.1.2; R Foundation). This activity was reviewed by CDC and conducted consistent with applicable federal law and CDC policy.^¶^

Among 770 pregnant persons who met inclusion criteria (17 with multiple gestations), 360 (46.8%) had a congenital syphilis pregnancy outcome (Table 1). Among 309 persons with a noncongenital syphilis pregnancy outcome and who did not use substances, 47.2% were aged <25 years, compared with 31.8% of those with a congenital syphilis pregnancy outcome who used substances. The prevalence of other age groups was distributed similarly across congenital syphilis pregnancy outcome and substance use status.

**TABLE 1:** Characteristics of pregnant persons with syphilis, by reported substance use* and congenital syphilis pregnancy outcome^†^ (N = 770) — Surveillance for Emerging Threats to Pregnant People and Infants Network, Arizona and Georgia, 2018–2021

Characteristic	No. (%)			
	Congenital syphilis (n = 360)		Noncongenital syphilis (n = 410)	
	Any substance use (n = 173)	No substance use (n = 187)	Any substance use (n = 101)	No substance use (n = 309)
**Age, yrs, median (IQR)**	27.7 (23.4–31.9)	26.3 (21.8–30.7)	27.8 (23.8–31.3)	25.3 (22.2–29.6)
**Age group, yrs**				
<25	55 (31.8)	72 (38.5)	37 (36.6)	146 (47.2)
25–29	59 (34.1)	63 (33.7)	31 (30.7)	92 (29.8)
30–34	37 (21.4)	34 (18.2)	25 (24.8)	54 (17.5)
≥35	21 (12.1)	16 (8.6)	8 (7.9)	17 (5.5)
Missing/Not reported	1 (0.6)	2 (1.1)	0 (—)	0 (—)
**Education level**				
Less than high school	51 (29.5)	55 (29.4)	30 (29.7)	94 (30.4)
High school graduate or GED	53 (30.6)	50 (26.7)	35 (34.7)	113 (36.6)
Some college but no degree	24 (13.9)	35 (18.7)	24 (23.8)	59 (19.1)
College degree or more	5 (2.9)	23 (12.3)	8 (7.9)	22 (7.1)
Missing/Not reported	40 (23.1)	24 (12.8)	4 (4.0)	21 (6.8)
**Insurance at delivery**				
Public	130 (75.1)	91 (48.7)	81 (80.2)	164 (53.1)
Private	12 (6.9)	12 (6.4)	6 (5.9)	31 (10.0)
Other/None/Self-pay	14 (8.1)	10 (5.3)	2 (2.0)	12 (3.9)
Missing/Not reported	17 (9.8)	74 (39.6)	12 (11.9)	102 (33.0)
**Prenatal care**				
First/Second trimester	78 (45.1)	124 (66.3)	79 (78.2)	281 (90.9)
Third trimester	26 (15.0)	25 (13.4)	13 (12.9)	16 (5.2)
No care	66 (38.2)	35 (18.7)	4 (4.0)	3 (1.0)
Missing/Not reported	3 (1.7)	3 (1.6)	5 (5.0)	9 (2.9)
No. of prenatal visits, median (IQR)	1 (0–6)	6 (1–10)	9 (6–11)	10 (7–13)
**Treatment**				
Adequate^§^	26 (15.0)	46 (24.6)	101 (100)	309 (100)
Inadequate^¶^	55 (31.8)	62 (33.2)	NA	NA
Not treated during pregnancy^¶^	92 (53.2)	79 (42.2)	NA	NA
History of incarceration**				
Yes	28 (16.2)	6 (3.2)	11 (10.9)	5 (1.6)
No	70 (40.5)	102 (54.5)	63 (62.4)	181 (58.6)
Missing/Not reported	75 (43.4)	79 (42.2)	27 (26.7)	123 (39.8)
History of homelessness**				
Yes	46 (26.6)	2 (1.1)	9 (8.9)	3 (1.0)
No	70 (40.5)	106 (56.7)	71 (70.3)	190 (61.5)
Missing/Not reported	57 (32.9)	79 (42.2)	21 (20.8)	116 (37.5)

**Abbreviations:** CSTE = Council of State and Territorial Epidemiologists; GED = general educational development certificate; NA = not applicable.

* Any substance use includes any use of tobacco (e.g., cigars, cigarettes, smokeless tobacco, or e-cigarettes), alcohol, cannabis, illicit use of opioids (e.g., prescription opioids not taken as prescribed, fentanyl, or heroin), and other illicit, nonprescription substances (e.g., cocaine, methamphetamines, inhalants, or hallucinogens, such as LSD or PCP).

^†^ Congenital syphilis pregnancy outcome includes pregnancy outcomes that meet the CSTE surveillance case definition for syphilitic stillborn or live-born infant with probable or confirmed congenital syphilis.

^§^ Adequacy of treatment dependent on syphilis stage. Primary, secondary, and early latent syphilis require at least 1 dose of penicillin during pregnancy, with the dose administered ≥30 days before pregnancy outcome. Late latent, latent of unknown duration, tertiary, and other cases of syphilis require ≥3 doses of penicillin, spaced 5–9 days apart, with the first dose administered ≥30 days before delivery and the final dose administered during pregnancy.

^¶^ Stillborn and live-born infants born to pregnant persons inadequately treated or not treated during pregnancy meet the CSTE case definition for a probable congenital syphilis pregnancy outcome.

** Within the 12 months preceding case report or positive test results or during pregnancy.

Among persons with a congenital syphilis pregnancy outcome, 53.2% of those who used substances and 32.1% of those who did not use substances received late (third trimester) or no prenatal care. Among persons with a noncongenital syphilis pregnancy outcome, 16.8% of those who used a substance and 6.1% of those who did not use a substance received late or no prenatal care. Irrespective of congenital syphilis pregnancy outcome, 39.8% of persons who used substances during pregnancy (274) either did not receive prenatal care or received it in the third trimester compared with 15.9% for those without substance use during pregnancy (496). Persons who used substances had, on average, six prenatal care visits, and those without substance use had nine. Among persons who used substances during pregnancy, 38.2% of those with a congenital syphilis pregnancy outcome received no prenatal care, compared with 4.0% of those with a noncongenital syphilis pregnancy outcome.

Among persons with a congenital syphilis pregnancy outcome, adequate treatment was received by 15.0% of those who did use any substances during pregnancy and 24.6% who did not. More than one half (53.2%) of 173 persons with a congenital syphilis pregnancy outcome and who used substances received no treatment for syphilis during pregnancy, compared with 42.2% of 187 persons who did not use substances.

Among persons who used substances during pregnancy, 16.2% of persons with a congenital syphilis outcome and 10.9% of persons with a noncongenital syphilis outcome had a history of incarceration; for history of homelessness in these groups the frequency was 26.6% and 8.9%. Data on incarceration were missing or not reported for 39% of all persons included in this analysis. Data on homelessness were missing or not reported for 35% of all persons included in this analysis.

Persons with a congenital syphilis pregnancy outcome were almost twice as likely to have used any substance during pregnancy as were those without this outcome (48.1% versus 24.6%; prevalence ratio [PR] = 1.95) (Table 2). Illicit use of opioids and illicit, nonprescription substances were the substance uses most frequently associated with a congenital syphilis pregnancy outcome. Illicit use of opioids during pregnancy was six times higher (PR = 6.09) and use of other illicit, nonprescription substances was more than four times higher (PR = 4.41) among persons with a congenital syphilis pregnancy outcome compared with those with a noncongenital syphilis outcome.

**TABLE 2:** Reported substance use*^,†^ among pregnant persons with syphilis, by congenital syphilis pregnancy outcome^§^ — Surveillance for Emerging Threats to Pregnant People and Infants Network, Arizona and Georgia, 2018–2021

Substance used	No. (%)		Prevalence ratio^¶^ (95% CI)
	Congenital syphilis (n = 360)	Noncongenital syphilis (n = 410)	
Any substance*	173 (48.1)	101 (24.6)	1.95 (1.60–2.38)
Tobacco	99 (27.5)	46 (11.2)**	2.45 (1.78–3.37)
Alcohol	29 (8.1)	20 (4.9)**	1.65 (0.95–2.86)
Cannabis	69 (19.2)	56 (13.7)^††^	1.40 (1.01–1.93)
Illicit use of opioids^§§^	75 (20.8)	14 (3.4)**	6.09 (3.50–10.58)
Illicit, nonprescription substance^¶¶^	101 (28.1)	26 (6.4)**	4.41 (2.94–6.63)

**Abbreviation:** CSTE = Council of State and Territorial Epidemiologists.

* Any substance use includes any use of tobacco (e.g., cigars, cigarettes, smokeless tobacco, or e-cigarettes), alcohol, cannabis, illicit use of opioids (e.g., prescription opioids not taken as prescribed, fentanyl, or heroin), and other illicit, nonprescription substances (e.g., cocaine, methamphetamines, inhalants, or hallucinogens such as LSD or PCP).

^†^ Numbers in categories are not mutually exclusive.

^§^ Congenital syphilis pregnancy outcome includes pregnancy outcomes that meet CSTE surveillance case definition for syphilitic stillborn and live-born infant with probable or confirmed congenital syphilis.

^¶^ Unadjusted.

** Denominator = 409.

^††^ Denominator = 408.

^§§^ Includes prescription opioids not taken as prescribed, fentanyl, and heroin.

^¶¶^ Includes other illicit, nonprescription substances (e.g., cocaine, methamphetamines, inhalants, or hallucinogens such as LSD or PCP).

## Discussion

Among pregnant persons in Arizona and Georgia, substance use prevalence was higher among those with a congenital syphilis pregnancy outcome than among those with a noncongenital syphilis outcome; the largest difference was observed in persons who used opioids illicitly or used other illicit, nonprescription substances. Consistent with previous research (*5*); the prevalence of late or no prenatal care was high among persons who used any substance during pregnancy, and those who did receive care had fewer prenatal visits. Prompt diagnosis and treatment of syphilis are critical to reducing adverse syphilis-related outcomes for persons who are pregnant, congenital syphilis, and overall syphilis transmission. The need for syphilis screening and treatment should be addressed at any health care encounter during pregnancy, especially among persons who use substances, and in all health care encounters with persons of childbearing age who have a high risk for syphilitic infection (*6*). Although syphilis is highly treatable with penicillin G (*5*,*7*), one third of persons in this analysis who used any substances remained untreated.

Previous studies suggest that social determinants of health, including incarceration and homelessness, might be associated with substance use and contribute to deficiencies in care and syphilis treatment (*5*,*8*). Although this study included small numbers and had high levels of missingness for history of incarceration and homelessness, up to one quarter of those who used substances and had a congenital syphilis pregnancy outcome had a history of incarceration or homelessness. Prioritizing persons with these lived experiences for screening and treatment of syphilis at every health care encounter is critical, and innovative strategies need to be developed to reach these populations.

The findings in this report are subject to at least five limitations. First, data collection is ongoing and is from only two states. Data from one of these states are restricted to only three counties; however, these counties represent approximately 80% of births in the state. Prevalence of substance use and other risk factors for congenital syphilis likely vary by jurisdiction, thereby limiting the generalizability of these results. Second, stigma and social desirability bias might have resulted in underreporting of substance use and contributed to the high missingness identified for history of incarceration and homelessness (*9*). Further, self-reported substance use creates the potential for recall bias by congenital syphilis status if captured retrospectively (after the birth) among those with a congenital syphilis pregnancy outcome. Fourth, because treatment is highly effective, the finding of 20% of persons with adequate treatment among those with congenital syphilis outcome could be an artifact of the CSTE case definition, which includes nonspecific clinical findings for probable cases or could be related to occult or undiagnosed reinfection that could not be assessed. Finally, there is no age limit for diagnosing congenital syphilis, which might create some misclassification in these data; however, almost all congenital syphilis cases are diagnosed during the neonatal period (*10*).

This report highlights the value of the SET-NET surveillance approach of linking data on pregnant persons to data on infants to understand factors related to congenital syphilis. The increasing numbers of congenital syphilis cases across the United States demand further exploration of factors that contribute to this trend and development of strategies to address missed opportunities for diagnosis and treatment before, during, and after pregnancy (*1*,*2*). Although screening and treatment can prevent most cases of congenital syphilis, numerous barriers to implementing these prevention strategies exist, some of which might be amplified among persons who use substances. Tailored interventions need to address barriers and facilitators for accessing screening and treatment for syphilis for persons with current or previous substance use, including those with a history of incarceration and homelessness.

**Summary**

**What is already known about this topic?**

Substance use prevalence has increased among women with syphilis; however, its association with congenital syphilis is less clear.

**What is added by this report?**

During 2018–2021, the prevalence of substance use among persons with syphilis during pregnancy in Arizona and Georgia was nearly twice as high among those with a congenital syphilis pregnancy outcome (48.1%) as among those without this outcome (24.6%). Approximately one half of persons who used substances during pregnancy and had a congenital syphilis pregnancy outcome had late or no prenatal care.

**What are the implications for public health practice?**

The need for syphilis screening and treatment should be addressed at every health care encounter during pregnancy, especially among persons using substances.

## Footnotes

*
https://ndc.services.cdc.gov/case-definitions/syphilis-2018/

† For Arizona, a pregnant person’s receipt of a positive syphilis test result and pregnancy outcome date occurred during 2019–2021. For Georgia, a pregnant person’s receipt of a positive syphilis test result occurred during 2017–2019 and pregnancy outcome date during 2018–2019.

§ Live-born infants were considered to have confirmed congenital syphilis if they met laboratory criteria for demonstration of *Treponema pallidum*. Live-born infants were considered to have probable congenital syphilis if the pregnant person had untreated or inadequately treated syphilis during pregnancy based on CDC treatment guidelines or if the infant received a reactive nontreponemal test result for syphilis and any of the following: evidence of syphilis on physical examination (excluding jaundice alone after 2019), abnormalities identified on long bone radiographs, reactive cerebrospinal fluid (CSF) venereal disease research laboratory test, or elevated CSF white blood cell counts or protein values. Stillborn infants were considered a syphilitic stillbirth if the pregnant person had untreated or inadequately treated syphilis during pregnancy based on CDC treatment guidelines and fetal death occurred after 20 weeks’ gestation or the fetus weighed >1.1 lbs (>0.5 kg).

¶ 45 C.F.R. part 46.102(l)(2), 42 U.S.C. Sect. 241(d); 5 U.S.C. Sect. 552a.
